# Development and Certification of a Reference Material for Aflatoxins and Zearalenone in Corn/Peanut Blended Vegetable Oil

**DOI:** 10.3390/foods14101667

**Published:** 2025-05-08

**Authors:** Jiaojiao Xu, Baifen Huang, Xiaomin Xu, Yiping Ren, Zengxuan Cai

**Affiliations:** Zhejiang Provincial Center of Disease Control and Prevention, Hangzhou 310051, China; jjxu@cdc.zj.cn (J.X.); bfhuang@cdc.zj.cn (B.H.); xmxu@cdc.zj.cn (X.X.); renyiping@263.net (Y.R.)

**Keywords:** blended vegetable oil, aflatoxins, zearalenone, certified reference material

## Abstract

A certified reference material (CRM) for aflatoxins (AFTB1, AFTB2, AFTG1, AFTG2) and zearalenone (ZEN) in corn/peanut blended vegetable oil (GBW(E)100863) was developed to address the critical need for matrix-specific reference materials in mycotoxin analysis. The CRM was prepared by blending naturally contaminated corn and peanut oils, followed by homogenization, sterilization, and sub-packing. Homogeneity and stability studies were conducted using high-performance liquid chromatography isotope-dilution tandem mass spectrometry with a dilute-and-shoot pretreatment process. The CRM demonstrated excellent homogeneity and stability, with no significant degradation observed under either short-term (65 °C for 14 days) or long-term (25 °C for 12 months) storage conditions. An inter-laboratory comparison involving six authoritative laboratories confirmed the CRM’s accuracy and reliability, with recovery rates ranging from 90.3% to 97.3% and low relative standard deviations (RSDs) of 3.79% to 7.99%. The CRM provided a robust metrological tool for mycotoxin analysis in complex oil matrices. This study not only enriches the national reference materials library but also supports food safety initiatives by facilitating accurate and reliable mycotoxin detection in vegetable oils, thereby enhancing regulatory compliance and public health protection.

## 1. Introduction

Reference materials (RMs), characterized by adequate homogeneity and stability, are essential metrological tools for ensuring comparable, reliable, and high-quality analytical results. They play a critical role in calibration, method validation, and quality control across various scientific and industrial fields. However, the current availability of RMs is limited in terms of matrix types and target concentrations, which often fail to meet the diverse demands of real-world testing scenarios. This limitation highlights the necessity to expand the variety of matrix types and concentration gradients of RMs, particularly in areas such as food safety, where accurate and precise measurements are paramount.

Vegetable oils, particularly blended oils, are essential components of a balanced diet due to their high content of unsaturated fatty acids and other bioactive compounds [1]. Peanut oil, for instance, is widely consumed for its pleasant aroma, digestibility, and nutritional benefits [2]. However, peanuts are highly susceptible to contamination by Aspergillus species, which produce aflatoxins, particularly aflatoxin B1 (AFTB1) [3]. Studies have shown that AFTB1 is a potent carcinogen, and its presence in peanut oil poses significant health risks [4,5]. Similarly, corn oil, derived from corn germ, is valued for its cholesterol-free composition and high levels of unsaturated fatty acids (80–85%), which contribute to the prevention of age-related diseases such as arteriosclerosis and diabetes [6]. Despite its nutritional benefits, corn oil is prone to contamination by aflatoxins and zearalenone (ZEN), primarily due to inadequate monitoring of raw materials during production [3,7]. Recent research has highlighted the advantages of blended vegetable oils over single-source oils, as blending can improve oxidative stability and nutritional profiles [1]. However, the risk of mycotoxin contamination remains a significant concern, as these toxins are not eliminated during the oil extraction process [3]. This has led to increased scrutiny of mycotoxin levels in edible oils and the need for robust analytical methods to ensure food safety.

In response to the health risks posed by mycotoxins, many countries have established regulatory limits for AFTB1, total aflatoxins, and ZEN in edible oils. For example, the European Union has set maximum limits of 2 µg/kg for AFTB1 and 4 µg/kg for total aflatoxins in vegetable oils [8]. Similarly, the U.S. Food and Drug Administration (FDA) has established action levels for aflatoxins in food products, including edible oils [9]. These regulations underscore the importance of accurate and reliable analytical methods for mycotoxin detection.

However, the analysis of mycotoxins in vegetable oils presents several challenges [10,11]. The complex matrix of oils can interfere with the detection of low-concentration mycotoxins, leading to potential inaccuracies. Additionally, the lack of standardized methods and reference materials for mycotoxin analysis in oil matrices complicates method validation and quality assurance. These challenges highlight the need for matrix-specific certified reference materials (CRMs) to improve the accuracy and comparability of analytical results. Despite the growing demand for reliable mycotoxin analysis, the availability of CRMs for vegetable oil matrices remains limited. Most existing CRMs focus on single mycotoxins or simpler matrices, such as cereals or nuts, which do not reflect the complexity of vegetable oils [4,12,13,14]. For instance, CRMs for AFTB1 in peanut butter or ZEN in cornmeal are commercially available, but their applicability to oil matrices is limited due to differences in matrix effects and extraction efficiency. Furthermore, there is a notable lack of CRMs for the aflatoxin G group and ZEN in vegetable oils, which are critical for comprehensive mycotoxin analysis. Recent studies have emphasized the importance of matrix-matched CRMs for accurate mycotoxin quantification. For example, Zhao et al. [15] developed a CRM for AFTB1 in peanut oil, demonstrating the feasibility of producing stable and homogeneous reference materials for complex oil matrices. However, their study was limited to a single mycotoxin and did not address the need for multi-mycotoxin CRMs. Similarly, our previous work (2018) [16] initially ensured that the AFTB1 has long-term stability and homogeneity in oil-based CRMs.

This study aims to address these gaps by developing a matrix CRM for multiple mycotoxins, including aflatoxins and zearalenone, in blended vegetable oil. The research will focus on assessing the homogeneity and stability (both short- and long-term) of the CRM to ensure its reliability for analytical applications. By providing a robust metrological tool, this work seeks to enhance the accuracy and comparability of mycotoxin analysis in vegetable oils, thereby supporting food safety initiatives and regulatory efforts.

## 2. Materials and Methods

### 2.1. Chemicals and Reagents

Pure aflatoxin B1 (AFTB1, 2.97 ± 0.09 µg/mL, product no. ERM-AC057), aflatoxin B2 (AFTB2, 2.98 ± 0.06 µg/mL, product no. ERM-AC058), aflatoxin G1 (AFTG1, 3.78 ± 0.13 µg/mL, product no. ERM-AC059), aflatoxin G2 (AFTG2, 2.98 ± 0.06 µg/mL, product no. ERM-AC060), and zearalenone (ZEN, 9.95 ± 0.30 µg/mL, product no. ERM-AC699) were obtained from the Institute for Reference Materials and Measurements (IRMM). The isotopes 13C-AFTB1, 13C-AFTB2, 13C-AFTG1, 13C-AFTG2, and 13C-ZEN in acetonitrile were purchased from Pribolab Pte. Ltd. (Qingdao, China). Certificated reference materials GBW(E)100386 (Aflatoxin B1 in maize) and GBW(E)100383 (zearalenone and deoxynivalenol in maize) were purchased from National Food and Strategic Reserves Administration (Beijing, China).

Methanol, acetonitrile, and formic acid were HPLC grade and purchased from Merck (Merck, Germany). Phosphate-buffer salts were AR grade and purchased from Pribolab Pte.Ltd.(Qingdao, China).

### 2.2. Selection of Candidate Reference Material

Vegetable oil samples were collected from retail markets throughout China, and comprehensive analyses were conducted to determine the presence of aflatoxins and zearalenone. These analyses aimed to identify naturally contaminated materials that could serve as raw reference materials. 

Following established regulatory limits [17] and food safety risk monitoring data from 2013 to 2022 in China, the AFTB1 and ZEN concentrations in the CRM were approximately defined as 10 µg/kg and 30 µg/kg, respectively. It plans to develop 500 packages of this reference material, each containing 40 grams. Firstly, the collected oil samples are filtered to remove obvious residue sediments and impurities, so as to avoid interfering substances. Subsequently, the concentrations of AFTB1, B2, G1, G2, and ZEN were measured in the collected peanut oil and corn oil samples. Initial results showed that the peanut oil contained AFTB1 at 75 µg/kg, AFTB2 at 12 µg/kg, AFTG1 at 0.88 µg/kg, AFTG2 at 0.22 µg/kg, and the corn oil contained ZEN at 160 µg/kg. Next, to create the blended vegetable oil CRM, precise quantities of the collected oils were combined. A total of 4 kg of aflatoxin-contaminated peanut oil, 5 kg of zearalenone-contaminated corn oil, 6 kg of aflatoxin-free peanut oil, and 5 kg of zearalenone-free corn oil were accurately weighed and mixed in a 1:1 ratio. This formulation resulted in a blended oil containing AFTB1, AFTB2, AFTG1, AFTG2, and ZEN as the target analytes. However, the naturally low concentrations of AFTG1 and AFTG2 in the collected oils were insufficient to meet the sensitivity requirements of various detection methods. Therefore, in this study, 1 mg each of AFTG1 and AFTG2 (purity >99%, purchased from Fermentek Ltd., Jerusalem, Israel) was weighed and dissolved in chloroform to prepare stock solutions with concentrations of 100 µg/mL. Subsequently, 0.8 mL of AFTG1 and 0.4 mL of AFTG2 stock solution were added to 20 kg of the blended vegetable oil. This fortification process achieved final concentrations of approximately 4 µg/kg for AFTG1 and 2 µg/kg for AFTG2 in CRM. The fortified blended oil was stirred at low speed for 72 h to ensure homogeneity and then packaged into brown glass bottles, sealed with aluminum foil, and stored at room temperature away from light. This packaging and storage protocol was designed to maintain the stability and integrity of the CRM during its shelf life.

### 2.3. Analytical Methods

The dilute-and-shoot method was employed in the preliminary experiments and certification. This method is widely recognized for its simplicity and efficiency in sample preparation [18]. The detailed steps are as follows: Accurately weigh 2 g of the sample (to an accuracy of 0.001 g) and place it in a 50 mL centrifuge tube. Add 8 mL of an acetonitrile–water solution (70:30, volume ratio) and mix for 1 min using a vortex mixer. Place the tube on a rotary shaker and extract by shaking for 30 min. Then, centrifuge at 8500 rpm for 10 min. Accurately transfer 0.5 mL of the supernatant to another 1.5 mL centrifuge tube. Add 1 mL of water, mix by vortex, and then centrifuge at 10,000 rpm for 10 min at 4 ℃. Aspirate the supernatant through a 0.22 μm filter membrane. Aspirate 180 μL of the processed sample filtrate into a 300 μL insert tube. Add 20 μL of the stable isotope mixed standard solution, mix, and prepare for LC-MS/MS analysis. 

Sample analysis was performed on a Waters Xevo TQ-S (Manchester, UK) LC-MS/MS, which was equipped with a Z-spray electrospray ionization (ESI) interface. Chromatographic separation was performed with an Acquity UPLC BEH C18 column (2.1 mm × 100 mm, 1.7 μm) at 40 ℃ column temperature. A flow rate of 0.3 mL/min was used, and the injection volume was set at 10 μL. The mobile phase consisted of solvent A (acetonitrile) and solvent B (water). The gradient elution program is listed as follows: 0 min∼1.5min, 15% A; 1.5 min∼6.5 min, 15%∼95% A; 6.5 min∼7.5 min, 95%A; 7.5 min∼8.0 min, 15% A. The mass spectrometer was operated with a source temperature of 150 ℃, a nebulizing gas of 150 L/h, a desolvation gas of 800 L/h, and a collision gas of 0.15mL/min. The detailed multiple reaction monitoring (MRM) parameters of the analytes are listed in Table 1.

Quantification of the target analytes (aflatoxins and zearalenone) was achieved using the stable isotope-labeled internal standards for calibration. The internal standards corrected for matrix effects and ensured accurate quantification. Data analysis was performed using Waters MassLynx software (V4.2), and the results were validated against established quality control criteria.

### 2.4. Comparative HPLC-FLD Analysis

Given that high-performance liquid chromatography with fluorescence detection (HPLC-FLD) is globally recognized as the benchmark method for aflatoxin analysis in regulatory settings, this study incorporated an immunoaffinity column (IAC) cleanup HPLC-FLD protocol as a supplementary approach. The implementation of this orthogonal methodology aimed to (i) verify the metrological traceability and cross-platform comparability of certified reference material (CRM) values and (ii) demonstrate the CRM’s applicability to routine analytical workflows widely adopted in food testing laboratories.

Chromatographic conditions: An ACQUITY UPLC I-Class system (Waters, Singapore) equipped with a large-volume flow-cell fluorescence detector was employed. Separation was achieved on an Acquity UPLC BEH C18 column (2.1 mm × 100 mm, 1.7 μm) maintained at 40°C, with a 10 μL injection volume. The mobile phase comprised methanol/acetonitrile (6:4, *v*/*v*; eluent A) and 0.1% formic acid (eluent B), with the following gradient profile at 0.3 mL/min: 0–1.5 min: 25% A; 1.5–6.5 min: 25–95% A; 6.5–7.5 min: 95% A; 7.5–8.0 min: 25% A. Fluorescence detection parameters were optimized at λex/λem = 274 nm/460 nm for ZEN and 365 nm/460 nm for aflatoxins.

Sample preparation: 2 g of the sample (to an accuracy of 0.001 g) were extracted with 8 mL acetonitrile/water (70:30, volume ratio) via 1 min vortex mixing followed by 30 min rotary shaking. After centrifugation (8500 rpm, 10 min), 2.0 mL supernatant was diluted with 25 mL PBS buffer (pH 7.2~7.4) to prepare the loading solution. Purification was performed using an Afla-ZEN 2-in-1 IAC (Pribonlab, Qingdao, China) with sequential PBS washing (5 mL), ultrapure water rinsing (5 mL), and methanol elution (3 mL). The eluate was nitrogen-evaporated (40°C), reconstituted in the initial mobile phase, and filtered prior to analysis.

### 2.5. Homogeneity and Stability Studies

Homogeneity and stability studies are critical steps to ensure the CRM’s reliability over its shelf life. To ensure the uniformity of the CRM, a random sampling method was employed. The experimental design for homogeneity testing is as follows: 500 aliquots of the candidate CRM were prepared, with each package containing approximately 40 g of the blended vegetable oil. Twenty bags were randomly selected from the 500 packages for homogeneity testing. Each selected bag was assigned a unique random number ranging from 1 to 20. Each bag was measured three times to assess intra-package variability. For each measurement, a minimum sample size of 2 g (accurate to 0.001 g) was taken from the bag.

To assess the short-term and long-term stability of the CRM, a split-group storage and testing approach was implemented. The experimental design for stability testing is as follows: Thirty bags of the prepared CRM were divided into 10 groups, with 3 bags in each group. Five groups were stored at 25 °C to simulate typical storage conditions for long-term stability. Testing was conducted on the day after sample preparation (Day 0) and after 1, 3, 6, and 12 months. The remaining five groups were stored at 65 °C to accelerate degradation and assess stability under stress conditions for short-term stability. Testing was conducted on the day after sample preparation (Day 0) and after 1, 3, 7, and 14 days. At each testing time point, one group was randomly selected from the stored groups. From each bag in the selected group, two samples were taken, and each sample was weighed at 2 g (accurate to 0.001 g).

### 2.6. Collaborative Value Assignment

To ensure the accuracy, reliability, and traceability of the CRM, a multi-laboratory collaborative value assignment approach was adopted. This approach involved the participation of six authoritative domestic testing institutions, each representing high-level detection capabilities in China. These laboratories must have extensive experience in the detection and analysis of mycotoxins in food, particularly aflatoxins and zearalenone. Laboratories were selected to represent diverse functional institutions, including disease control centers, quality inspection agencies, and customs laboratories, to ensure broad applicability of the CRM in regulatory and supervisory contexts. Possessing different brands of LC-MS/MS instruments, laboratories were randomly assigned two CRM samples, with each sample measured at least six times, yielding a minimum of 12 data points per laboratory. Participants used the same or similar validated methods, ensuring consistency while allowing methodological diversity. For quality control, ERM-series certified reference materials and maize powder CRMs (GBW(E)100386/GBW(E)100383) were used, alongside spike recovery validation to assess method accuracy.

### 2.7. Statistical Analysis

Statistical analyses were conducted using Microsoft Excel. Results were expressed as means ± standard deviation (SD) to provide a measure of central tendency and variability. One-way analysis of variance (ANOVA) was used to assess intra- and inter-package variability. This parametric test is robust for comparing means across multiple groups. The *F*-test within ANOVA determined whether observed variations were statistically significant, with a *p*-value < 0.05 indicating significance. For long-term and short-term stability data, regression analysis was performed to evaluate trends over time. A *p*-value < 0.05 in regression analysis indicated significant changes in analyte concentrations, suggesting instability. Data from multiple laboratories were combined to calculate consensus values and their associated uncertainties. Grubbs’ test was applied to detect and remove outliers, ensuring the reliability of the dataset. The mean, SD, and confidence intervals were calculated to provide a comprehensive summary of the collaborative study results.

## 3. Results

### 3.1. Optimization of Dilute-and-Shoot Linked with LC-MS/MS in Determination for Aflatoxins and Zearalenone in Blended Vegetable Oil

The extraction efficiencies of two solvent systems—acetonitrile–water (70:30, volume ratio) and acetonitrile–water–formic acid (70:29:1, volume ratio)—were evaluated for the target mycotoxins (aflatoxins B1, B2, G1, G2, and zearalenone). Recovery rates (*n* = 3) are shown in Table 2. The acetonitrile–water (70:30) system yielded recovery rates ranging from 90.3% to 97.3% across all target mycotoxins. The acetonitrile–water–formic acid (70:29:1) system showed slightly lower recovery rates, ranging from 85.3% to 93.2%. The addition of formic acid did not result in an increase in recovery rates across all analytes. Thus, the acetonitrile–water (70:30) system was utilized in determinations.

The research team also examined the timing of isotope internal standard addition, comparing the impact of incorporating the isotope internal standard at the onset of extraction versus adding it solely prior to injection (*n* = 3), with the results subjected to *t*-test analysis. As presented in Table 3, the *p*-values obtained from the *t*-test were all greater than 0.05, indicating that there was no significant difference between the two approaches to isotope internal standard addition. Consequently, it is feasible to add the isotope internal standard only before injection to account for matrix effects in liquid chromatography tandem mass spectrometry.

The matrix effect (ME) of this method was evaluated based on the mathematical model: ME = (Peak area of the spiked matrix extract (SET 1) − Peak area of the matrix extract (SET 2))/Peak area of the standard solution at the same spiked concentration (SET 3), i.e., ME = (SET 1 − SET 2)/SET 3. An ME value of 1 indicates no matrix effect; ME > 1 indicates a matrix enhancement effect; and ME < 1 indicates a matrix suppression effect. Peanut oil and corn oil samples, devoid of AFTs and ZEN, were selected and mixed in a 1:1 volume ratio. The blank matrix sample solution was prepared by extraction, dilution, and filtration according to the pre-treatment method. Accurately transfer 1 μL of the mixed standard stock solution (0.5 μg/mL) into 999 μL of the blank matrix sample solution, and mix thoroughly to obtain the sample matrix-matched standard solution. Accurately transfer 1 μL of the mixed standard stock solution (0.5 μg/mL) into 999 μL of a 15% acetonitrile–water solution, and mix thoroughly to obtain the pure standard solution. Samples were injected sequentially, and the injection was repeated twice to obtain the mean peak area, with the results presented in Table 4. The peak areas for SET 1 (spiked matrix extract) and SET 3 (pure standard solution) were comparable for all target analytes. SET 2 (matrix extract without spiking) was not applicable, as no peaks were detected for the blank matrix. The results demonstrate that ME is close to 1, indicating that this method exhibits minimal matrix effects, with no significant enhancement or suppression observed.

**Figure 1:**
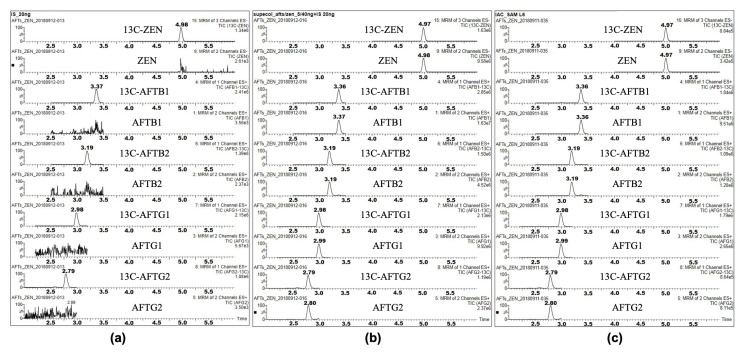
MRM chromatogram of target compounds and their internal standard compounds in (**a**) isotope standard solution, (**b**) standard mixture solution, and (**c**) CRM.

To ensure the accuracy and reliability of the quantification method, the research group employed 13C-labeled isotope internal standards for aflatoxins (AFTB1, B2, G1, G2) and ZEN. A critical step in this process was to verify that the isotope internal standards did not introduce interference with the target compounds. Multiple reaction monitoring (MRM) channels specific to the isotope internal standards were examined for any chromatographic peaks at the retention times corresponding to the target compounds. No significant chromatographic peaks were observed at the retention time positions of the target compounds (AFTB1, AFTB2, AFTG1, AFTG2, and ZEN) in the MRM channels of the isotope internal standards (as illustrated in Figure 1). This confirms that the 13C-labeled isotope internal standards do not interfere with the detection and quantification of the target analytes.

### 3.2. Homogeneity Assessment

The homogeneity of the candidate reference materials was assessed in accordance with ISO 33405-2024 [19]. The data were subjected to analysis of variance (ANOVA) to evaluate the variability within and between units. The results are summarized in Table 5. The *F*-values for all target compounds (AFTB1, AFTB2, AFTG1, AFTG2, and ZEN) were lower than the critical *F*-values at a 95% confidence level (α = 0.05), indicating no significant differences between units. This confirms that the candidate materials are homogeneous for aflatoxins and zearalenone at the 95% confidence level.

The uncertainty contribution due to potential heterogeneity ($u_{homo}$) was calculated using Equations (1) and (2), based on the mean squares within bottles (*MS_within_*) and between bottles (*MS_between_*).

(1) $$u_{homo}=\sqrt{\frac{{MS}_{between}-MS_{within}}{n}}$$

(2) $$u_{homo}^{*}=\sqrt{\frac{{MS}_{within}}{n}}\cdot \sqrt[{4}]{\frac{2}{N-m}}$$

whereas $u_{homo}$ represents inhomogeneity estimate for *MS_between_* > *MS_within_*, $u_{homo}^{*}$ represents inhomogeneity estimate for *MS_between_* < *MS_within_*, *n*, *m* and *N* are number of replicate sub-samples per unit (here *n* = 3), number of units (here *m* = 20) and number of total detected samples for homogeneity study (here *N* = 60), respectively. The larger value of $u_{homo}$ or $u_{homo}^{*}$ was used as the uncertainty contribution for homogeneity.

To evaluate the minimum sample size required for homogeneity assessment in blended corn/peanut vegetable oil, a statistical analysis using analysis of variance (ANOVA) was conducted. Four sample units were randomly selected, and each unit was sampled twice at different sample sizes: 0.5 g, 1 g, and 2 g. The homogeneity and within-unit standard deviations were examined for each sample size. At a sample size of 0.5 g, ZEN exhibited significant differences between sample units, indicating potential inhomogeneity. At sample sizes of 1 g and 2 g, no statistically significant differences (*p* > 0.05) were observed for any of the target compounds (AFTB1, AFTB2, AFTG1, AFTG2, and ZEN), confirming homogeneity. Besides, the within-unit standard deviation was analyzed to assess variability at different sample sizes. At a sample size of 1 g, the within-unit variability was significantly higher compared to that at 2 g for all target compounds. This indicates that a sample size of 2 g provides more consistent and reliable results, with reduced variability.

### 3.3. Stability Report

The short-term and long-term stability of the target compounds (AFTB1, AFTB2, AFTG1, AFTG2, and ZEN) in blended corn/peanut vegetable oil were evaluated under simulated transport (65 °C for 7 days) and storage conditions (25 °C for 12 months). The results were analyzed using linear regression based on ISO 33405-2024 to assess the significance of changes in compound concentrations over time.

The slope (*b*_1_) of the regression line, representing the relationship between mass fraction and time, was calculated for each compound. The significance of the slope was evaluated using the *t*-test, where *b*_1_/*S_b_*_1_ (the ratio of the slope to its standard error) was compared to the critical *t*-value (*t*_0.95, *n*−2_ = 2.16) at a 95% confidence level. For all target compounds, *b*_1_/*S_b_*_1_ was less than the critical *t*-value, indicating that the slopes were not statistically significant (see Table 6). This confirms that the compounds remained stable under both short-term (65 °C for 7 days) and long-term (25 °C for 12 months) conditions.

The uncertainty contributions from short-term stability (*u_sts_*) and long-term stability (*u_lts_*) were calculated using Equation (3):

(3) $$u_{lts}\;or\;u_{sts}=\frac{{RSD}_{stab}}{\sqrt{\sum {\left(x_{i}-\bar{x}\right)}^{2}}}\cdot t\;$$

where *RSD*_stab_ is the relative standard deviation of stability study results, *x_i_* is the time point for each replicate, $\bar{x}$ is the average of all time points, *t* is the pre-defined shelf life (7 days for short-term stability and 12 months for long-term stability).

Since no degradation was observed under either condition, the combined uncertainty from both short-term and long-term stability was considered in the overall uncertainty evaluation.

### 3.4. Certification Study and Value Assignment

Six laboratories, including both governmental and non-governmental organizations, applied the dilute-and-shoot strategy for the analysis of aflatoxins and ZEN. The purity and uncertainty of the mycotoxin standards were obtained from the provider. Prior to data processing, a technical and statistical evaluation of the submitted data was conducted. All recovery values for the target compounds fell within the range of 90.6% to 114.0%, meeting the regulatory requirements specified in EC 401/2006 [20] for mycotoxins with content below 1 mg/kg. No datasets were excluded due to technical outliers. Datasets were assumed to be normally distributed and evaluated for outliers using Dixon’s and Grubbs’ tests at a significance level of α = 0.05. No outliers were detected. The Cochran test (at a 95% confidence level) was used to assess the agreement among analytical methods. No outliers were identified for the five target mycotoxins. The consensus values for aflatoxins and zearalenone were calculated as the mean of laboratory means, providing appropriate estimates for the mass fractions. Therefore, the average value calculated from the detected values by six laboratories was adopted as the certified value of multi-mycotoxin CRMs in peanut oils. Resultantly, the certified values for aflatoxins and ZEN in blended oil CRMs were listed in Table 7.

The uncertainty in the value assignment process, involving multiple laboratories, is evaluated through type A and type B uncertainty assessments. Type A uncertainty (*u_A_*) is calculated as the standard deviation of the mean values from *m* laboratories, each performing *n* measurements. The uncertainty contributions were calculated using Equation (4):

(4) $$u_{A}=t_{\alpha }(m-1)\times \sqrt{\sum_{i=1}^{m}{\left(\bar{X_{i}}-\bar{X}\right)}^{2}/m(m-1)}$$

where $\bar{X_{i}}$ is the mean from each laboratory, $\bar{X_{i}}$ is the overall mean, and *t*_α_ (*m* − 1) is the *t*-value at significance level α.

**Figure 2:**
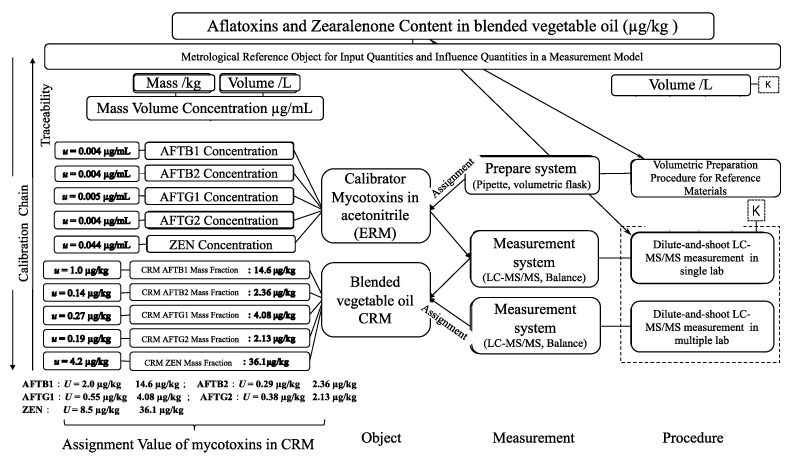
Traceability chart for blended vegetable oil CRM.

Type B uncertainty (*u_B_*) evaluation involves analyzing each step of the measurement process. Based on the traceability chart (Figure 2), potential sources of uncertainty affecting the measured component are identified. The combined standard uncertainty *u_B_* is calculated as Equation (5):

(5) $$u_{B}=\sqrt{u_{S}^{2}+u_{IS}^{2}+u_{SP}^{2}+u_{c}^{2}+u_{m}^{2}+u_{m\_V}^{2}+u_{IS\_V}^{2}}$$

where *u_s_* and *u_Is_* is the uncertainty introduced by certified reference materials and corresponding isotope internal standard, *u_sp_* are the uncertainties introduced during the preparation of mixed standard stock solutions and working solutions, *u_c_* is the standard uncertainty introduced during the calibration curve fitting process, *u_m_* is the standard uncertainty introduced during sample weighing, *u_m_V_* and *u_IS_V_* is the standard uncertainty introduced by the volume of sample extraction and the volume addition of the internal standard working.

### 3.5. Uncertainty Evaluation

The certified value’s uncertainty comprises three components: homogeneity ($u_{homo}$), stability (short-term stability (*u_sts_*) and long-term stability (*u_lts_*)), and characterization (*u_A_* and *u_B_*). These components are combined using Equation (6) to obtain the combined standard uncertainty of the reference material (*u_CRM_*):

(6) $$u_{CRM}=\sqrt{u_{char}^{2}+u_{homo}^{2}+u_{s}^{2}}=\sqrt{u_{A}^{2}+u_{B}^{2}+u_{homo}^{2}+u_{lts}^{2}+u_{sts}^{2}}$$

The expanded uncertainty ($U_{CRM}$) of the reference material’s characteristic value is obtained by multiplying the combined standard uncertainty $u_{CRM}$ by a coverage factor *k* (where *k* = 2 for a 95% confidence level). The summary of the uncertainty components for aflatoxins (AFT B1, AFT B2, AFT G1, AFT G2) and zearalenone (ZEN) is presented in Table 8.

## 4. Discussion

The development and certification of a CRM for aflatoxins (AFTB1, AFTB2, AFTG1, AFTG2) and ZEN in blended corn/peanut vegetable oil represent a significant advancement in the field of food safety and mycotoxin analysis. This study not only addresses the critical need for matrix-specific CRMs but also provides a comprehensive evaluation of the analytical methods, stability, and collaborative value assignment, ensuring the reliability and traceability of the CRM for use in food safety monitoring and regulatory compliance.

The method, based on high-performance liquid chromatography isotope-dilution tandem mass spectrometry, was optimized and validated for accurate mycotoxin quantification in the blended vegetable oil. The dilute-and-shoot approach, combined with isotope-labeled internal standards, was chosen for its simplicity and efficiency in sample preparation [15]. The recovery rates for the target mycotoxins ranged from 90.3% to 97.3%, which is within the acceptable range for regulatory purposes, demonstrating the method’s robustness and suitability for complex oil matrices. The evaluation of ME revealed minimal interference, with ME values close to 1, indicating neither significant enhancement nor suppression of the target analytes. This finding is crucial, as matrix effects are a common challenge in mycotoxin analysis, particularly in complex matrices like vegetable oils [11]. The use of isotope-labeled internal standards further mitigated matrix effects, ensuring accurate quantification and method reliability. The timing of isotope internal standard addition was also investigated, and the results showed no significant difference between adding the internal standard at the onset of extraction or prior to injection, simplifying the sample preparation process without compromising accuracy.

The stability of the CRM was thoroughly evaluated under both short-term (65 °C for 14 days) and long-term (25 °C for 12 months) storage conditions. The results of the stability studies, analyzed using linear regression, showed no significant degradation of the target mycotoxins over time. The slopes of the regression lines for all analytes were not statistically significant (*p* > 0.05), confirming the stability of the CRM under the tested conditions. This stability is critical for ensuring the CRM’s reliability over its shelf life, particularly for its use in long-term analytical applications and quality control. The short-term stability test at 65 °C, which simulates accelerated degradation conditions, further validated the CRM’s robustness under stress conditions. The absence of significant degradation under these conditions suggests that the CRM can withstand potential temperature fluctuations during transportation and storage, which is essential for its practical application in laboratories worldwide.

The certification of the CRM involved a multi-laboratory collaborative study, with six authoritative domestic laboratories participating in the value assignment process. This collaborative approach ensured the accuracy, reliability, and traceability of the CRM’s certified values. Each laboratory performed at least six measurements per sample, yielding a minimum of 12 data points per laboratory. The use of validated methods and quality control measures, such as spike recovery validation and the use of certified reference materials, further enhanced the reliability of the results. Consensus values for aflatoxins and ZEN were calculated as the mean of laboratory means, with relative standard deviations (RSDs) ranging from 3.79% to 7.99%. These low RSDs indicate good agreement among the participating laboratories, confirming the CRM’s homogeneity and the robustness of the analytical methods used. The uncertainty associated with the certified values was evaluated through type A and type B uncertainty assessments, considering contributions from homogeneity, stability, and the characterization process. The combined standard uncertainty (*u_CRM_*) for the target mycotoxins ranged from 0.14 µg/kg to 4.2 µg/kg, with expanded uncertainties (*U_CRM_*) at a 95% confidence level (*k* = 2) ranging from 0.29 µg/kg to 8.5 µg/kg.

To further validate the applicability of the CRM across diverse analytical platforms, the accuracy and reliability of the certified values were cross-verified using the HPLC-FLD method, a widely adopted method in regulatory laboratories. As demonstrated in Table 9, the results from IAC-HPLC-FLD exhibited excellent agreement with the certified values derived from isotope-dilution LC-MS/MS. The relative differences between methods were consistently below 5%, confirming the CRM’s robustness across analytical techniques. This dual-method validation underscores the CRM’s versatility in supporting laboratories utilizing either fluorescence-based or mass spectrometry-based workflows. By ensuring traceability and compatibility with both routine (HPLC-FLD) and advanced (LC-MS/MS) methods, this CRM addresses the critical need for matrix-matched reference materials capable of harmonizing multi-mycotoxin analysis in complex oil matrices.

The study builds on our previous work, which developed a reference material for AFTB1 in peanut oil. It goes further by including multiple mycotoxins (AFTB1, AFTB2, AFTG1, AFTG2, and ZEN) in a blended vegetable oil matrix, which more closely reflects real-world scenarios where multiple mycotoxins may co-occur. This multi-mycotoxin approach is particularly important given the increasing regulatory scrutiny of mycotoxin levels in edible oils and the need for comprehensive analytical methods. The inclusion of ZEN, a mycotoxin with estrogenic effects, further broadens the applicability of the CRM, as ZEN contamination in vegetable oils has been increasingly reported in recent years [21,22]. Moreover, the study addresses the limitations of existing CRMs, which are often limited to single mycotoxins or simpler matrices [13,23,24,25]. The development of a CRM for a blended vegetable oil matrix, which is more complex and representative of real-world food products, represents a significant step forward in mycotoxin analysis. The study also highlights the importance of matrix-matched CRMs for accurate mycotoxin quantification, as differences in matrix effects and extraction efficiency can significantly impact analytical results [26].

Furthermore, this CRM enriches China’s national reference materials library, serving as a valuable resource for researchers, regulators, and the food industry. It also sets a precedent for the development of additional matrix-specific CRMs for other mycotoxins and food matrices, which will further enhance the robustness of mycotoxin analysis globally. The CRM’s certification by six authoritative domestic laboratories ensures its broad applicability in regulatory and supervisory contexts, making it a valuable tool for ensuring food safety and compliance with national and international standards. This is particularly important in light of the stringent regulatory limits for mycotoxins in edible oils set by organizations such as the European Union, the FDA, and China.

## 5. Conclusions

The successful development and certification of this CRM (GBW(E)100863) for aflatoxins (AFTB1: 14.6 ± 2.0 µg/kg, AFTB2: 2.36 ± 0.29 µg/kg, AFTG1: 4.08 ± 0.55 µg/kg, AFTG2: 2.13 ± 0.38 µg/kg) and zearalenone (ZEN: 36.1 ± 8.5 µg/kg) in blended vegetable oil represent a critical advancement in mycotoxin analysis. The CRM demonstrated robust metrological performance. Its homogeneity and stability (no degradation at 65 °C/14 days or 25 °C/12 months) ensure reliability for long-term applications. Cross-verified using both HPLC-FLD and LC-MS/MS methods confirmed methodological consistency (relative differences <5%), enabling broad utility in laboratories employing diverse analytical workflows. By addressing the scarcity of matrix-specific CRMs for multi-mycotoxin analysis in oils, this work directly supports global food safety initiatives, enhances regulatory compliance, and provides a traceable foundation for harmonized mycotoxin monitoring in complex matrices.

## Figures and Tables

**Table 1:** Multiple reaction monitoring (MRM) parameters of the analytes in blended vegetable oil.

Compound	Parent Ion (*m*/*z*)	Daughter Ion (*m*/*z*)	Collision Energy (eV)	Cone Voltage (V)	Ion Model
AFT B1	313	285 */241	20/35	37	ESI+
AFT B2	315	287 */259	24/30	42	ESI+
AFT G1	329	243 */214	20/28	36	ESI+
AFT G2	331	313 */245	24/30	36	ESI+
ZEN	317	175 */131	24/30	44	ESI−
[13C]-AFT B1	330	301	20	37	ESI+
[13C]-AFT B2	332	303	24	42	ESI+
[13C]-AFT G1	346	257	28	36	ESI+
[13C]-AFT G2	348	330	24	36	ESI+
[13C]-ZEN	335	185	26	42	ESI−

Note: * for quantitative ion.

**Table 2:** Comparison of recovery rates for two extracted solvents (*n* = 3).

Recovery Average%	AFTB1	AFTB2	AFTG1	AFTG2	ZEN
acetonitrile–water	95.6	97.3	94.4	91.1	90.3
acetonitrile–water–formic acid	93.2	88.8	91.2	86.4	85.3

**Table 3:** Comparison of isotope internal standard addition timing (*n* = 3).

Detective Results (μg/kg)	AFTB1	AFTB2	AFTG1	AFTG2	ZEN
onset of extraction	14.4	2.49	3.90	1.99	39.3
prior to injection	14.7	2.31	3.96	2.04	39.4
*p* Value	0.131	0.060	0.207	0.275	0.474

**Table 4:** Matrix effect evaluation for target analytes.

Area	AFTB1	AFTB2	AFTG1	AFTG2	ZEN
SET 1	114698	76334	103352	51337	18983
SET 2	/	/	/	/	/
SET 3	114887	70044	98051	47372	17864
ME	1.00	1.09	1.05	1.08	1.06

**Table 5:** ANOVA table for homogeneity study of aflatoxins in candidate materials and estimates for uncertainty contribution.

Analyte	Con. (μg/kg)	Mean Squares (MS, μg/kg)		*F*_value_	*F*_critial_ (α = 0.05)	*u_homo_*
		*MS_between_* (*m* = 20)	*MS_within_* (*m* = 3)			
AFTB1	14.5	0.5033	0.2816	1.79	1.85	0.30
AFTB2	2.24	0.0342	0.0271	1.26		0.05
AFTG1	4.27	0.0232	0.0128	1.81		0.06
AFTG2	2.21	0.0118	0.0082	1.44		0.03
ZEN	35.9	6.8294	3.7877	1.80		1.01

**Table 6:** Results of short-term and long-term stability studies.

Analyte	65 °C (Short-Term Study)			25 °C (Long-Term Study)		
	Slope (*b*_1_)	*b*_1_/*S_b_*_1_	Significance	Slope (*b*_1_)	*b*_1_/*S_b_*_1_	Significance
AFTB1	−0.01	0.18	No	0.07	1.32	No
AFTB2	0.002	0.41	No	−0.002	0.58	No
AFTG1	0.01	0.81	No	0.001	0.08	No
AFTG2	−0.002	0.68	No	−0.004	0.32	No
ZEN	0.09	0.54	No	0.23	0.81	No

**Table 7:** Assigned value of reference material certified by multiple laboratories.

Analyte	AFTB1	AFTB2	AFTG1	AFTG2	ZEN
Average	14.6	2.36	4.08	2.13	36.1
Standard deviation (SD)	0.8	0.10	0.30	0.17	1.4
Relative Standard deviation (RSD)%	5.44	4.21	7.46	7.99	3.79

**Table 8:** Summary of uncertainty values for aflatoxins and zearalenone.

Value	AFTB1	AFTB2	AFTG1	AFTG2	ZEN
*u_A_* (µg/kg)	0.2	0.03	0.09	0.05	0.4
*u_B_* (µg/kg)	0.02	0.01	0.01	0.01	0.1
*u_homo_* (µg/kg)	0.3	0.05	0.06	0.03	1.0
*u_sts_* (µg/kg)	0.6	0.07	0.13	0.05	2.3
*u_lts_* (µg/kg)	0.6	0.05	0.18	0.13	3.4
*u_CRM_* (µg/kg)	1.0	0.14	0.27	0.19	4.2
*U_CRM_* (µg/kg, *k* = 2)	2.0	0.29	0.55	0.38	8.5

**Table 9:** Cross-verified for CRM by HPLC-FLD and LC-MS/MS methods.

Value ± Uncertainty (μg/kg)	AFTB1	AFTB2	AFTG1	AFTG2	ZEN
HPLC-FLD (single lab)	14.7 ± 1.0	2.36 ± 0.22	4.26 ± 0.31	2.19 ± 0.26	37.7 ± 3.7
LC-MS/MS (multiple labs)	14.6 ± 2.0	2.36 ± 0.29	4.08 ± 0.55	2.13 ± 0.38	36.1 ± 8.5

## Data Availability

The original contributions presented in the study are included in the article; further inquiries can be directed to the corresponding author.
